# Centre‐level variation of treatment and outcome in 5‐year‐old children with non‐syndromic unilateral cleft lip and palate: The Cleft Care UK study. Part 1: Methodology and results for dento‐facial outcomes

**DOI:** 10.1111/ocr.12183

**Published:** 2017-06-29

**Authors:** A. K. Wills, O. Mahmoud, A. Hall, D. Sell, J. Smallridge, L. Southby, S. Toms, A. Waylen, Y. Wren, A. R. Ness, J. R. Sandy

**Affiliations:** ^1^ National Institute for Health Research (NIHR) Biomedical Research Unit in Nutrition, Diet and Lifestyle The University Hospitals Bristol NHS Foundation Trust and the University of Bristol Bristol UK; ^2^ Bristol Dental School University of Bristol Bristol UK; ^3^ School of Social and Community Medicine University of Bristol Bristol UK; ^4^ Life and Health Sciences Aston University Birmingham UK; ^5^ Children’s Hearing Centre St Michael’s Hospital Bristol UK; ^6^ North Thames Regional Cleft Service, Speech and Language Therapy Department Centre for Outcomes and Experience Research in Children's Health, Illness and Disability (ORCHID) Great Ormond Street Hospital NHS Foundation Trust London UK; ^7^ Cleft Net East Cleft Network Addenbrooke's Hospital Cambridge UK; ^8^ Bristol Speech Language Therapy Research Unit North Bristol NHS Trust Bristol UK; ^9^ Department of Applied Statistics Helwan University Cairo Egypt

**Keywords:** centralization, cleft lip and palate, treatment outcomes, variation

## Abstract

**Objectives:**

Outline methods used to describe centre‐level variation in treatment and outcome in children in the Cleft Care UK (CCUK) study. Report centre‐level variation in dento‐facial outcomes.

**Setting and Sample Population:**

Two hundred and sixty‐eight five‐year‐old British children with non‐syndromic unilateral cleft lip and palate (UCLP).

**Materials and Methods:**

Between January 2011 and December 2012, data were collected on a comprehensive range of outcomes. Child facial appearance and symmetry were assessed using photographic pictures. Dental arch relationships were assessed from standardized dental study models. Hierarchical statistical models were used to predict overall means and the variance partition coefficient (VPC)—a measure of amount of variation in treatment or outcome explained by the centre.

**Results:**

Data on dento‐alveolar arch relationships and facial appearance were available on 197 and 252 children, respectively. The median age of the children was 5.5 years, and 68% were boys. Variation was described across 13 centres. There was no evidence of centre‐level variation in good or poor dento‐alveolar arch relationships with a VPC of 4% and 3%, respectively. Similarly, there was no evidence of centre‐level variation in good or poor facial appearance with a VPC of 2% and 5%, respectively.

**Conclusions:**

There was no evidence of centre‐level variation for dento‐facial outcomes although this study only had the power to detect large variation between sites.

## 1 INTRODUCTION

Care for children born with a cleft of the lip and/or palate in the United Kingdom is now centralized and multidisciplinary^1^, and outcomes have improved.^2^, ^3^, ^4^, ^5^, ^6^, ^7^ These changes have been implemented since the Clinical Standards Advisory Group (CSAG) reported in 1998.^8^ A key recommendation was that the number of centres offering cleft services should be reduced from 57 to approximately 8 to 15. Since the report, the number of centres was reduced to eleven managed clinical networks. Care in these centres is now provided by multidisciplinary teams, and the surgeons in these teams operate on at least 35 cases each year.^1^

We conducted the Cleft Care UK (CCUK) study fifteen years later to evaluate the impact of these changes to care.^4^ CCUK is a national cross‐sectional survey of the treatment and outcomes of 5‐year‐old children born with UCLP that used a comparable design with similar measurements to a previous survey.^4^ We reported the results in detail in a previous supplement of this journal.^2^, ^3^, ^4^, ^5^, ^6^, ^7^ Briefly, treatment had changed—the range of surgical procedures used was less varied, hearing aids were used more often and grommets placed less frequently; and some outcomes had improved—dentoalveolar relations and speech were considerably better, but the prevalence of dental caries and hearing loss was unchanged. Although the changes were generally positive, comparative data suggest that outcomes for children in the UK are still not as good as the best centres in Europe.^9^, ^10^, ^11^ Furthermore, a significant proportion of children still experienced poor outcomes within this centralized service.^3^

In this supplement, we explore centre‐level variation in treatment and outcomes and predictors of outcomes for children UCLP treated within this centralized multidisciplinary service. We hope that describing and exploring this variation may identify further areas where care and outcomes can be improved. In this study, we outline the approach we have adopted to describe centre‐level variation and illustrate this by reporting the results for facial growth (dento‐alveolar relationship) and facial appearance. Subsequent papers in this supplement employ the same approach to describe centre‐level variation in audiology,^12^ oral health,^13^ speech^14^ and behavioural outcomes.^15^ The final paper summarizes the results of these analyses and discusses implications.^16^

## 2 METHODS

### 2.1 Study sample

We used data from CCUK. This is a UK‐wide cross‐sectional study of 5‐year‐old children born between April 2005 and March 2007 with UCLP. A full description of recruitment procedures and eligibility criteria can be found elsewhere.^4^ Briefly of 359 eligible children, consent for participation was obtained from 268 (75%) children and parents.

### 2.2 Cleft centres and managed clinical networks

We collected data from every cleft centre in the UK (n=18) at designated audit clinics. Several of the centres are managed by a single Clinical Director as part of a clinical network or hub. For example, the centres Liverpool and Manchester are part of the North West and North Wales network, and Bristol and Swansea are part of the South West and South Wales network. These 18 centres function within 11 managed clinical networks. The full list of centres and their associated clinical network is shown in Table 1.

**Table 1:** Organization of cleft services in the UK at the time of the study

Clinical network	Centre
Cleft Net East	Cambridge
West Midlands	Birmingham
Northern & Yorkshire	Leeds
	Newcastle
Northern Ireland	Belfast
North Thames	Great Ormond Street
	Chelmsford
North West & North Wales	Liverpool
	Manchester
Scotland	Glasgow
	Edinburgh
	Aberdeen
South Thames	Guys & St Thomas’
South West & South Wales	Bristol
	Swansea
Spires	Salisbury
	Oxford
Trent	Nottingham

### 2.3 Outcomes

We report on dento‐alveolar relationship and facial appearance in this paper. Full details of the methodology for these two variables have been reported elsewhere.^4^ Dental arch relationships were assessed from standardized casts of impressions taken of each child's teeth and mouth. The models were assessed using the established 5‐Year‐Olds' Index, creating an ordinal response on a five‐point Likert‐type scale (1=Excellent, 2=Good, 3=Fair, 4=Poor or 5=Very Poor). Facial appearance was assessed from photographs using a standardized and validated aesthetic outcome assessment tool for the evaluation of cleft lip and palate surgical repairs. An orthodontist, blinded to the centre, rated each cropped image using a 5‐point Likert‐type scale (1=Excellent, 2=Good, 3=Fair, 4=Poor or 5=Very Poor). This five‐point ordinal scale was adapted and developed by the Birmingham Institute of Paediatric Plastic Surgery from an existing method.^17^

### 2.4 Co‐variables

Differences in age and gender may have effects on developmental outcomes such as facial structure and speech, so age and gender were considered as potential confounders of centre‐level differences. Although the target age was between 5 years 3 months and 5 years 9 months, the mean age in the study was 5 years and 7 months and the range was 4 years 6 months to 7 years and 6 months. The cleft centres scheduled special audit clinics when the children were the required age. However, where appointments were missed, clinics had to be rescheduled and children would then have been seen at different ages.

### 2.5 Coding of outcomes

Both of the outcome variables in this study are ordinal. To focus the results on the between‐centre variation in good outcomes and the between‐centre variation in poor outcomes, each variable was recoded into two binary variables. To capture the good outcomes, for both variables, children scored as a one (excellent) or two (good) were coded as one with all others zero; and to capture the poor outcomes, children scored as a four (poor) or five (very poor) were coded as one with all others zero.

### 2.6 Statistical model

Hierarchical models (also known as multilevel, mixed‐effects or random‐effects models) were fitted to each of the binary outcome variables. Centre was treated as a random effect, and sex and age were modelled as fixed binary and continuous covariates. A logit‐link function was used (ie, logistic) giving the following model.

$$y_{ij}∼Binomial\left(1,\pi_{ij}\right)$$

$$logit\left(\pi_{ij}\right)=\beta_{0}+\beta_{1}\;age+\beta_{2}\;sex+\mu_{j}$$

(1) $$\mu_{j}∼N\left(0,\sigma_{\mu }^{2}\right)$$

In the model above (Equation 1), *y*_*ij*_ is the binary response (0 or 1) for child *i* in centre *j*, $\sigma_{\mu }^{2}$ is the between‐centre variance in the outcome adjusting for age and sex differences, *β*_0_ is the log odds of *y* in girls at age 5.5 years (age was centred at the median of 5.5 years and boys were coded as 1) when *μ*_*j*_ = 0 (i.e, in the so‐called average centre). Lastly, *μ*_*j*_ is an estimate of the difference in log odds of having the outcome (*y *= 1) between centre *j* and the so called average centre *β*_0_. The models were estimated by maximizing the likelihood function using numerical integration (adaptive Gauss‐Hermite approximation). In cases where estimation of $\sigma_{\mu }^{2}$ failed, we used (a) a quasi‐likelihood method using penalized iteratively reweighted least squares^18^ and if (a) failed then (b) Bayesian estimation via Markov Chain Monte Carlo methods.^19^ We used R (vers 3.3.2), and the lme4, blme and R2MLwiN packages package to estimate the hierarchical models. Stata (vers 14.2) was used for all other analyses.

### 2.7 Describing variation between centres

The variance partition coefficient (VPC) was estimated from these models as a measure of the variability in outcomes between centres. The VPC captures the proportion of the total variation in each outcome that is explained by differences between hubs. A VPC has the same interpretation as an intraclass correlation coefficient (ICC), and in certain models, they are the same. In our models, which contain the fixed covariates age and sex, the VPC can also be interpreted as the residual correlation between individuals from the same centre. As fixed covariates were included in the model, we used a simulation approach to estimate the VPC (http://www.bristol.ac.uk/cmm/software/support/support-faqs/pval.html#b). A likelihood ratio test of the null hypothesis that there is no variation between hubs (ie, $\sigma_{\mu }^{2}=0$) was also performed.

### 2.8 Estimating mean levels for treatment and outcome for each centre

We used $\beta_{0}$ to estimate the proportion of children with the outcome in the so‐called average centre. As age and sex were included in the models, $\beta_{0}$ represents the predicted log odds of the outcome for girls of median age 5.5 years. The equation in (1) was rearranged to convert $\beta_{0}$ from logs odds to a proportion $\left(\pi_{ij}\right)$. In a similar way,$\beta_{0}+\mu_{j}$ was used to predict the proportion of children in each centre with each outcome. These predicted proportions are model‐based estimates of the mean in each centre and are more likely to be closer to the true value than a simple description of the raw data because they borrow information from the overall mean in a way that is proportional to the sample size in each centre and to the amount of clustering or similarity in outcomes from individuals within each centre.

## 3 RESULTS

### 3.1 Sample description and grouping of centres

The median age of children in CCUK was 5.5 years (IQR: 5.4‐5.7 years), and 181 of 268 (68%) were boys. Data on dento‐alveolar arch relationships and facial appearance were available on 197 and 252 children, respectively. Table 2 shows the number of children treated in each centre. As the sample size was small for particular centres, we grouped centres by their managed clinical networks except for those centres that had treated more than 15 children. This resulted in 13 centres all of which had more than 15 children (Table 2).

**Table 2:** Number of children (n) treated in each centre and n in final grouping of centres used in the analyses (ordered by centre size)

	n (%) in each centre	n (%) in each centre/hub unit
1	3 (1.1)	16 (6.0)
2	6 (2.2)	16 (6.0)
3	8 (3.0)	16 (6.0)
4	10 (3.7)	17 (6.3)
5	10 (3.7)	17 (6.3)
6	11 (4.1)	19 (7.1)
7	13 (4.9)	19 (7.1)
8	13 (4.9)	20 (7.5)
9	13 (4.9)	21 (7.8)
10	16 (6.0)	23 (8.6)
11	16 (6.0)	24 (9.0)
12	16 (6.0)	30 (11.2)
13	17 (6.3)	30 (11.2)
14	17 (6.3)	
15	19 (7.1)	
16	20 (7.5)	
17	30 (11.2)	
18	30 (11.2)	
Total	268 (100)	268 (100)

### 3.2 Age and sex distribution by centres

There was evidence of an association between cleft centre and age at assessment (*P*<.001). However, the mean age of the children in each centre was within 2 months of the overall mean for all centres except one. The single centre outside of this limit assessed children that were on average more than 6 months older than the mean. There was also evidence of an association between the sex of the child and centre (*P*=.017), although the variation was small—in all but one centre, more boys were assessed than girls.

### 3.3 Centre‐level variation in dento‐alveolar relations

**Figure 1:**
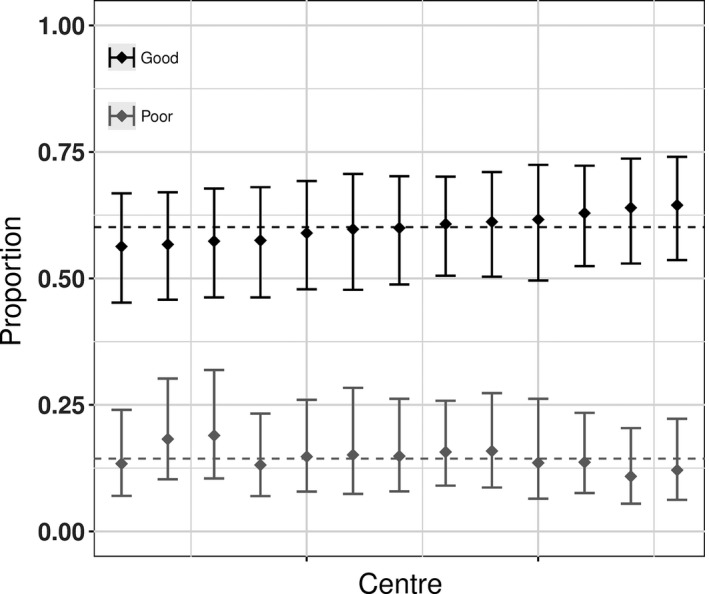
Predicted proportion in each centre with a good outcome (black) and a poor outcome (grey) for dento‐alveolar relationship. The bars are 95% confidence intervals, and the dashed line is the predicted mean for the average centre adjusted for age and sex

Table 3 shows the results for the good and poor dento‐alveolar relationship outcomes. The overall percentage of children with a good outcome and with a poor outcome in so‐called average centres was 60% (95% CI: 48%‐71%) and 14%, respectively (95% CI: 7%‐28%). There was no evidence for any centre‐level variation in these outcomes—after adjusting for age and sex, only 2% and 4% of the variability in good and poor outcomes, respectively, was attributable to differences between centres. Figure 1 shows the predicted proportions with good and poor dento‐alveolar relationships for each centre. It is clear that outcomes were broadly similar across all centres.

**Table 3:** Predicted proportions with each outcome for the so‐called “average” centre and the between‐centre variability variance partition coefficient (VPC)

Outcome		n	Proportion (95% CI)	VPC	*P*‐value^a^
Dento‐alveolar	Good (1 or 2)	197	0.6 (0.48, 0.71)	0.04	.6
	Poor (4 or 5)		0.14 (0.07, 0.28)^b^	0.03	.9
Facial appearance	Good (1 or 2)	252	0.37 (0.24, 0.52)^b^	0.02	.9
	Poor (4 or 5)		0.18 (0.09, 0.34)^b^	0.05	.9

All results are adjusted for age and sex.

a a test of the null hypothesis that there is no between‐centre variation.

b Due to small estimated level 2‐variance $\sigma_{\mu }^{2}<0.001$, the CI was estimated using Bayesian approach by maximizing model's posteriori.

### 3.4 Centre‐level variation in facial appearance

**Figure 2:**
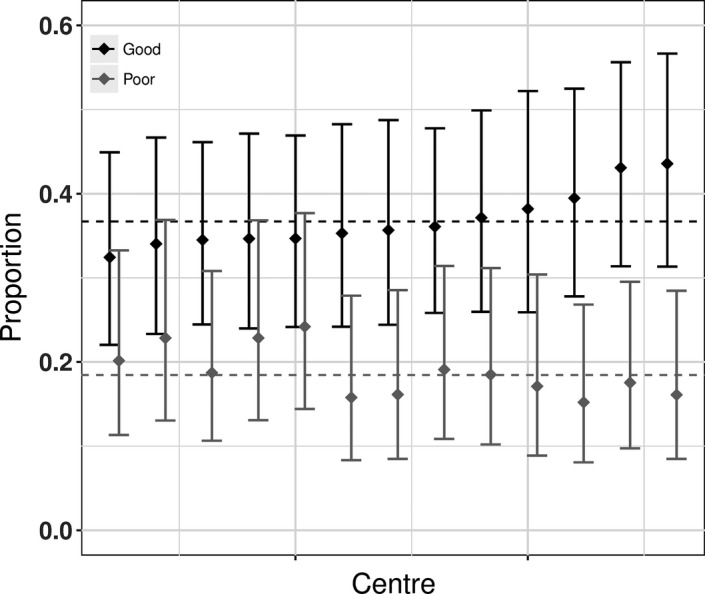
Predicted proportion in each centre with a good outcome (black) and a poor outcome (grey) for facial appearance. The bars are 95% confidence intervals, and the dashed line is the predicted mean for the average centre. Adjusted for age and sex

Table 3 shows the results for the good and poor facial appearance outcomes. The overall percentage of children with a good outcome and with a poor outcome in so‐called “average” centres was 37% (95% CI: 24%‐52%) and 18%, respectively (95% CI: 9%‐34%). As with dento‐alveolar relations, there was no evidence of centre‐level variation for these outcomes. After adjusting for age and sex, only 3% and 5% of the variability in good and poor outcomes, respectively, was attributable to differences between treatment centres. Figure 2 shows the predicted proportions for each centre. It is clear that outcomes did not vary between centres beyond what would be expected from random sampling variation.

## 4 DISCUSSION

We have described a standard hierarchical statistical model to predict centre‐level means and centre‐level variation and highlighted how to interpret the output from these models using the expository examples of facial growth and appearance. We found modest differences between centres in the age at assessment and gender of the children over and above that which may be expected from sampling variation and so we controlled for these differences in the analysis presented in this paper and in the subsequent papers in this supplement.^12^, ^13^, ^14^, ^15^ We found no evidence of centre‐level variation in dento‐alveolar relationships and facial appearance.

### 4.1 Strengths and limitations

Cleft Care UK is a large study (for children with cleft lip and palate), nationwide and contains a substantial set of validated measures of key outcomes measured with enough precision to demonstrate improvements over time.^2^, ^3^, ^4^, ^5^, ^6^, ^7^ The response rate was also good. Nonetheless, our study has limited power to detect modest centre‐level variation in treatment and outcome, so our results with respect to centre‐level variation need to be interpreted with caution. We tried to minimize this risk by pooling smaller centres with their respective regional network.

### 4.2 The importance of exploring centre‐level variation

In this supplement, we have explored centre‐level variability across a range of treatment and outcome measures. Our results, which are reported in full in each respective paper in this supplement,^12^, ^13^, ^14^, ^15^, ^16^ suggest that there was centre‐level variation in several of these treatment indicators and outcomes across the domains of oral health,^13^ audiology^12^ and speech.^14^ Describing and exploring these centre‐level variations is important for several reasons. First, it may describe differences in treatment and outcome between centres that can be explored and that may ultimately result in improvements in care and outcome. Second, centre may act as a confounder of certain association. Third, if there is a correlation between individuals from the same centre or clustering of observations then the assumption of independent observations that is made for conventional statistical analysis does not hold. This leads to invalid inference through standard errors that are estimated to be too small. The multilevel models used here deal with these issues.

It is important to remember the work that led to the changes in cleft services in the UK which raised expectations with regard to potential influences to improve outcomes. Seminal work in the late 1970s in Europe (known as the Eurocleft study) showed between‐centre variation in outcomes irrespective of protocols or surgical techniques.^20^ Six centres submitted consecutive UCLP cases treated within a given time period. When dento‐alveolar outcomes were examined, the centre with the worst results had multiple surgeons and no standardized policy for surgery. The efficacy of individual elements of different treatment programmes could not be detected by examining dento‐alveolar outcomes. As cleft services have centralized in the UK, there is evidence that variations in surgical techniques are narrowing which would explain, in part, limited centre differences in this outcome.^21^ The Eurocleft study also examined soft tissues using both cephalometrics and naso‐labial appearance.^22^, ^23^ Similar outcome rankings were seen across the six centres whether using dento‐alveolar, cephalometric or nasolabial measures. However, sample size variations between centres and the “coarseness” of these measures limited identification of factors which contributed to the good or poor outcomes.

In the UK, data on outcomes from the CSAG study originated from 57 centres which included dento‐alveolar relations and naso‐labial appearance.^24^, ^25^ None of these cleft centres had sufficient cases to enable meaningful comparison with other centres or indeed the overall national outcomes. Post‐centralization of cleft care, the number of cases that each surgeon treats per year has increased. In 2009‐2010, seventeen of the nineteen primary cleft surgeons in the UK operated on 40‐50 cases annually,^1^ whereas only a single surgeon achieved this in the original CSAG.^25^ Arguments were made as to what would be a volume of surgery sufficient to detect differences between centres, surgeons and techniques. Even if one restricts follow‐up to 5 years, only operators treating 60 new cases per year would be able to audit their outcomes within a decade.^26^ This was highlighted in a recent well‐designed adequately powered study.^27^ Three parallel randomized clinical trials were undertaken as an international multicentre study by 10 cleft teams in five countries: Denmark, Finland, Sweden, Norway and the UK. All children included were born with UCLP and randomized to three different surgical procedures for primary palatal repair. There was no evidence that one technique is better than the others used. The possible influence of individual surgical skill was recognized.^27^ This factor was not analysed in the current study where individual surgical volumes preclude meaningful analysis.

### 4.3 Centre‐level variation in dento‐alveolar relationships and facial appearance

Although outcomes for dento‐alveolar relationships and facial appearance have improved with the introduction of a centralized multidisciplinary service^2^ there was no evidence of variation between centres within this centralized service. There are several reasons for this. First, this study only had the power to detect large differences so it is possible that modest but important differences do exist between centres but our study did not have the power to detect these. Second, it is possible that these outcomes are not measured accurately enough to detect modest centre‐level differences—although we used standard photographic protocols and approaches to coding, facial appearance is difficult to measure accurately and reliably. Furthermore, intraoral photographs were used in some children rather than study models in some centres to assess dento‐alveolar relationships. We have previously shown that reliability of scoring is reduced for intraoral photographs compared with dental study casts and that on the currently available evidence, dental study casts still provide the gold standard when assessing primary surgical outcome in cleft care.^28^ Thus, the use of intraoral photographs instead of dental study casts may have reduced variation in assessing dento‐alveolar relationships between centres. Third, it is possible that variation between surgeons rather than centres is important in determining these outcomes.^27^ Finally, it is possible that care is now so uniform in this centralized service that variations in these outcomes between centres do not exist.^21^

## 5 CONCLUSION

Children with cleft lip and palate in the UK are now treated by a centralized multidisciplinary service that has resulted in improved outcomes. Variation in treatment and outcomes between centres may still exist within this centralized service. Describing and exploring centre‐level differences in treatment and outcome may help improve care and outcomes for children with cleft lip and palate. We found no evidence of centre‐level variation in dento‐alveolar arch relationship and facial appearance.

## APPENDIX 1

### List of local study PIs, Co‐ordinators and staff who contributed to the collection of data for the CCUK project

**Table:**

Cleft Centre	
Cleft NetEast, Cambridge	Per Hall (PI); Sue Burgess (Co‐ordinator)
West Midlands, Birmingham	Rona Slator (PI); Alexander Levine (Co‐ordinator) Victoria Clark; Lars Enocson; Alison Jeremy
South Wales South West, Bristol	Liz Albery (PI); Richard Willerton
North Thames, Great Ormond Street Hospital for Children	Loshan Kangesu (PI); Laura Sennett (Co‐ordinator) Norman Hay; Karen Wilson; Raouf Chorbachi; Marie Pinkstone; Natalie Pancewicz; Anne Mayne; Brijesh Patel; James Green; Debbie Sell; Laura Sennett; Lauren Baillie; John Volcano
South Thames, Guys and St Thomas’	Alex Cash (PI); Peggy Mo (Co‐ordinator)
Spires, Oxford	Stephen Robinson (PI); Steven Berry (Co‐ordinator)
Northern and Yorkshire, Leeds	Alistair Smyth (PI); Heather Jamieson (Co‐ordinator)
South Wales South West, Swansea	Adrian Sugar (PI); Andrea Thomas (Co‐ordinator)
Trent, Nottingham	John Rowson (PI); Vicky Nightingale (Co‐ordinator)
Royal Aberdeen Children's Hospital	Felicity Mehendele (PI)
West of Scotland, Glasgow	Toby Gilgrass (PI); Elaine Simpson (Co‐ordinator)
East of Scotland, Edinburgh	Felicity Mehendele (PI)
Northwest England, Isle of Man, North Wales Liverpool	Joyce Russell (PI); Helen McCormick (Co‐ordinator)
Royal Manchester Children's Hospital	Haydn Bellardie (PI); Claire Richardson (Co‐ordinator) Jeanette Mooney, Jill Painter, Melanie Bowden, Julie Davies, Jayne O'Connor, David Whitby, Jenny Williams, Vicky Beale.
Northern and Yorkshire, Newcastle	Peter Hodgkinson (PI); Paula Spence (Co‐ordinator)
Spires, Salisbury	Stephen Robinson (PI); Mary Ann Brewer (Co‐ordinator)
North Thames, St. Andrew's Centre, Chelmsford	Loshan Kangesu (PI); Karen Wilson (Co‐ordinator)
Northern Ireland	Chris Hill (PI); Pamela Foster (Co‐ordinator)
